# Differences in insect resistance between tomato species endemic to the Galapagos Islands

**DOI:** 10.1186/1471-2148-13-175

**Published:** 2013-08-24

**Authors:** Alejandro F Lucatti, Adriaan W van Heusden, Ric CH de Vos, Richard GF Visser, Ben Vosman

**Affiliations:** 1 Wageningen UR Plant Breeding, Wageningen University and Research, Centre, P.O. Box 386, Wageningen, AJ 6700, The Netherlands; 2 Graduate School Experimental Plant Sciences. Wageningen Campus. Droevendaalsesteeg 1, 6708 PB, Wageningen, The Netherlands; 3 Plant Research International, Business Unit Bioscience, Wageningen University and Research Centre, P.O. Box 619, Wageningen, AP 6700, The Netherlands; 4 Centre for BioSystems and Genomics, P.O. Box 98, Wageningen, AB 6700, The Netherlands; 5 Netherlands Metabolomics Centre, Einsteinweg 55, 2333 CC, Leiden, The Netherlands

**Keywords:** *Bemisia tabaci*, *Solanum galapagense*, *Solanum cheesmaniae*, Whitefly, Trichomes, Acyl sugars, Selection pressure

## Abstract

**Background:**

The Galapagos Islands constitute a highly diverse ecosystem and a unique source of variation in the form of endemic species. There are two endemic tomato species, *Solanum galapagense* and *S*. *cheesmaniae* and two introduced tomato species, *S*. *pimpinellifolium* and *S*. *lycopersicum*. Morphologically the two endemic tomato species of the Galapagos Islands are clearly distinct, but molecular marker analysis showed no clear separation. Tomatoes on the Galapagos are affected by both native and exotic herbivores. *Bemisia tabaci* is an important introduced insect species that feeds on a wide range of plants. In this article, we address the question whether the differentiation between *S*. *galapagense* and *S*. *cheesmaniae* may be related to differences in susceptibility towards phloem-feeders and used *B*. *tabaci* as a model to evaluate this.

**Results:**

We have characterized 12 accessions of *S*. *galapagense*, 22 of *S*. *cheesmaniae*, and one of *S*. *lycopersicum* as reference for whitefly resistance using no-choice experiments. Whitefly resistance was found in *S*. *galapagense* only and was associated with the presence of relatively high levels of acyl sugars and the presence of glandular trichomes of type I and IV. Genetic fingerprinting using 3316 SNP markers did not show a clear differentiation between the two endemic species. Acyl sugar accumulation as well as the climatic and geographical conditions at the collection sites of the accessions did not follow the morphological species boundaries.

**Conclusion:**

Our results suggest that *S*. *galapagense* and *S*. *cheesmaniae* might be morphotypes rather than two species and that their co-existence is likely the result of selective pressure.

## Background

Tomatoes are native to South America and can be found from the north of Chile/Argentina to Ecuador, including the Galapagos Islands. The Galapagos is a volcanic archipelago of 13 islands located about 1000 km from the coast of Ecuador. On this archipelago, vegetation varies among islands, altitude and cardinal direction [1,2]. The Galapagos Islands constitute a highly diverse ecosystem and a unique source of variation in the form of endemic species. There are two endemic tomato species, *Solanum galapagense* and *S*. *cheesmaniae* and two introduced species *Solanum pimpinellifolium* and *S*. *lycopersicum*. It is believed that the latter two have been introduced on the islands during the twentieth century, though it is possible that *S*. *pimpinellifolium* is present on the islands a lot longer [1]. The endemic Galapagos Island tomato species have evolved in isolation from the mainland species, resulting in clearly differing morphological features compared to the species that were introduced later. However, natural hybrids have been found [1,3]. The taxonomic status of the Galapagos’ endemic tomatoes is under debate and a historic overview is given by Darwin *et al*. (2003). Darwin and co-workers adopted the ‘morphological cluster’ species concept [4] to divide the two endemic tomato forms in two species *S*. *galapagense* and *S*. *cheesmaniae*. Despite the clear separation obtained on the basis of morphology, it was not possible to separate the two species with molecular markers [2,5-7].

From the total number of endemic species on the Galapagos Islands, 47% are insects [8]. However, the number of exotic insect species on the Galapagos is increasing due to human activity and now at least 463 exotic insect species can be found on the archipelago, of which 193 species are herbivores and in majority phloem feeders [9]. Of the exotic insect species 73% are naturalized or are known to feed on endemic plant species. The whitefly *Bemisia tabaci* (Gennadius) (Hemiptera: Aleyrodidae) is one of the most important invasive insects on the Galapagos Islands, receiving the highest score of invasiveness due to their wide distribution, wide host range and their importance as vector of many plant viruses [9-11]. Recently it became clear that *B*. *tabaci* is not a single species but a whole complex of at least 36 cryptic species [12-15]. The first report of *B*. *tabaci* on the Galapagos dates back to 1998 [9]. Interestingly, *S*. *galapagense* was shown to be resistant to this devastating pest insect [14,16,17]. This suggests that a general mechanism conferring resistance towards insects is present in *S*. *galapagense* as the time after introduction of *B*. *tabaci* is too short for co-evolution. The resistance is associated with the presence of type IV glandular trichomes [14,17].

Selection pressure, co-evolutionary processes, population genetics, bio-geographical variables, and gene dynamics (gene flow, drift, mating systems, etc.) can affect the evolution of resistance genes and the accumulation or the presence of certain metabolites related to resistance [18,19]. For example, the evolution of genes involved in the terpenoid pathway and the relative composition of acyl sugar were suggested to be related to geographic and climatic variables under which populations of *Solanum habrochaites* are found [20,21]. The Galapagos Islands have proven to be a place of natural experimental conditions to answer questions related to processes like founder effect, genetic drift, divergent selection, ecological opportunity and interaction between endemic and exotic species in the speciation processes [8]. As *B*. *tabaci* has an ecological importance on the Galapagos Islands [9] and its global importance as a phloem feeding tomato herbivore, we decided to use *B*. *tabaci* as a model insect to analyse factors underlying insect resistance in relation to species delimitation between *S*. *galapagense* and *S*. *cheesmaniae*.

So far, only a limited number of accessions/populations of *S*. *galapagense* and *S*. *cheesmaniae* have been evaluated for insect resistance and therefore it is unknown if the insect resistance coincides with the species boundaries (based on the morphological differences). Neither is there any knowledge about the relation between geographical and climatic conditions today on the Galapagos and the occurrence of the two species. Recently it was shown that the whitefly resistance in an accession of *S*. *galapagense* is most likely based on the production of acyl sugars in the glandular trichomes [22], it is unknown if the relative acyl sugar concentration among the different accessions of *S*. *galapagense* and *S*. *cheesmaniae* coincides with species boundaries and insect resistance. In the present study, we address the questions raised and discuss the implication in an evolutionary context. We characterized the genetic and acyl sugar variation in 34 endemic tomato accessions and investigated if resistance and chemical variation among accessions are correlated. We demonstrate that *S*. *galapagense* is different form *S*. *cheesmaniae* in the resistance towards whiteflies and in the trichome composition. Geographic and climatic variables do not explain the distribution pattern found for the Galapagos’ endemic tomatoes. Genetic variation between the two species is almost absent and acyl sugar composition does not completely follow the morphological species boundaries. All together our results suggest that *S*. *galapagense* and *S*. *cheesmaniae* might be considered as morphotypes rather than two species and that their co-existence is likely the result of selective pressure.

## Results

### Resistance to whitefly

The level of whitefly resistance in accessions of *S*. *galapagense*, *S*. *cheesmaniae* and cv. Moneymaker was assessed using three parameters, namely adult survival (AS), oviposition rate (OR), and pre-adult survival (PS) (Table 1). The three parameters were highly correlated (Table 2). For AS, significant differences were found among accessions (ANOVA, p < 0.001), with survival rates ranging from 0 to 1. The lowest values for AS were found within the accessions of *S*. *galapagense*, with five accessions on which all whiteflies were dead after 5 days (AS = 0). Adult survival on all accessions of *S*. *galapagense* was statistically different from the AS on cv. Moneymaker. None of the *S*. *cheesmaniae* accessions were statistically different from cv. Moneymaker for the resistance variables (Table 1). When comparing AS at the species level, *S*. *galapagense* was the most resistant species (AS = 0.09 ± 0.12, p < 0.001).

**Table 1:** **Adult survival, oviposition rate and pre-adult survival of the different accessions of *Solanum galapagense *and *S*. *cheesmaniae***

**Taxa and accession no.**	**Adult survival**			**Oviposition rate**			**Pre-adult survival**		
	**n**	**mean**		**n**	**mean**		**n**	**mean**	
*Solanum cheesmaniae*		0.89			7.04			0.59	
LA0421	(5)	0.99	*kl*	(5)	6.02	*de*	(3)	0.46	*def*
LA0422	(9)	0.89	*hij*	(9)	6.25	*de*	(5)	0.83	*h*
LA0428	(9)	0.69	*gh*	(9)	6.06	*de*	(5)	0.41	*de*
LA0521	(2)	0.98	*ijkl*	(2)	8.74	*e*	(2)	0.63	*efgh*
LA0522	(8)	0.89	*ijkk*	(8)	5.78	*de*	(5)	0.52	*efg*
LA0528B	(9)	0.85	*hij*	(9)	8.40	*e*	(5)	0.71	*gh*
LA0529	(2)	0.50	*hij*	(2)	5.50	*cde*	(1)	0.48	ND
LA0746	(9)	0.86	*hi*	(9)	6.01	*de*	(5)	0.66	*gh*
LA0932	(6)	0.94	*ijkl*	(6)	7.43	*de*	(2)	0.70	*fgh*
LA1035	(4)	0.97	*ijkl*	(4)	7.33	*de*	(0)	ND	ND
LA1039	(3)	1.00	*l*	(2)	7.11	*de*	(1)	0.35	ND
LA1040	(9)	0.93	*ijkl*	(9)	6.36	*de*	(4)	0.77	*gh*
LA1041	(4)	0.86	*hij*	(4)	7.19	*de*	(0)	ND	ND
LA1042	(9)	0.94	*ijkl*	(9)	8.21	*e*	(5)	0.65	*fgh*
LA1043	(8)	0.95	*ijkl*	(8)	8.77	*e*	(3)	0.74	*gh*
LA1137 ^#^	(9)	0.94	*ijkl*	(9)	7.72	*e*	(5)	0.75	*gh*
LA1139	(4)	1.00	*l*	(4)	6.62	*de*	(3)	0.51	*efg*
LA1404	(9)	0.90	*ijkl*	(9)	7.59	*de*	(5)	0.66	*fgh*
LA1409	(2)	0.75	*ghi*	(1)	5.45	ND	(1)	0.01	ND
LA1411	(10)	0.95	*ijkl*	(10)	8.65	*e*	(0)	ND	ND
LA1450	(9)	0.91	*ijkl*	(9)	8.86	*e*	(4)	0.64	*fgh*
LA3124	(6)	0.97	*jkl*	(6)	4.76	*cd*	(6)	0.76	*gh*
*Solanum galapagense*		0.09			0.99			0.12	
LA0438	(3)	0.00	*ab*	(2)	0.00	*a*	(0)	ND	ND
LA0480A	(7)	0.29	*def*	(7)	2.89	*bc*	(3)	0.07	*abc*
LA0483	(3)	0.00	*ab*	(3)	0.00	*a*	(0)	ND	ND
LA0528	(9)	0.22	*cde*	(9)	2.86	*bc*	(5)	0.10	*bc*
LA0530	(5)	0.10	*abc*	(5)	0.20	*a*	(1)	0.50	ND
LA0532	(3)	0.00	*ab*	(3)	0.17	*a*	(0)	ND	ND
LA0748	(8)	0.30	*ef*	(8)	2.48	*bc*	(3)	0.23	*cd*
LA1401	(8)	0.03	*ab*	(7)	0.57	*a*	(1)	0.00	ND
LA1408	(6)	0.00	*a*	(6)	0.00	*a*	(0)	ND	ND
LA1452	(5)	0.00	*ab*	(5)	0.14	*a*	(2)	0.00	*a*
LA1508	(8)	0.11	*bcd*	(8)	1.90	*b*	(4)	0.02	*abc*
LA1627	(9)	0.04	*ab*	(9)	0.70	*a*	(2)	0.02	*ab*
*Solanum lycopersicum*									
Cv. Moneymaker	(33)	0.93	*ijkl*	(33)	5.55	*de*	(15)	0.51	*efg*

n: Number of plants per accession.

#: Classified as *S*. *galapagense* in TGRC, but phenotypically it is a *S*. *cheesmaniae*.

ND: Not determined.

Different letters indicate statistical differences according to LSD test (p < 0.05). *S*. *lycopersicum* cv. Moneymaker is included as a reference.

**Table 2:** Pearson correlation among phenotypic resistance characteristics and Acyl sugar accumulation

	**Resistance variables**			**Trichome type**					**Acyl sugar**
	**AS**	**OR**	**PS**	**I**	**III**	**IV**	**V**	**VI**	
AS		**	**	**	0.13	**	**	0.02	**
OR	1.00		**	**	0.12	**	**	0.02	**
PS	0.81	0.82		**	0.06	**	**	0.16	*
Trichome type I	-0.92	-0.92	-0.84		0.06	**	**	0.07	**
Trichome type III	0.33	0.34	0.39	-0.39		0.07	*	0.19	0.07
Trichome type IV	-0.92	-0.92	-0.84	1.00	-0.39		**	0.06	**
Trichome type V	0.88	0.88	0.73	-0.91	0.55	-0.90		0.37	**
Trichome type VI	-0.50	-0.48	-0.30	0.39	0.28	0.40	-0.20		0.13
Total Acyl sugar	-0.61	-0.60	-0.51	0.64	-0.38	0.64	-0.57	0.33	

Below main diagonal is the correlation coefficient and upper the main diagonal is the *p* value associated. Asterisk (*) indicate p ≤ 0.01, double asterisks (**) indicate p ≤ 0.001.

The oviposition rate (OR) ranged from 0 to 8.86 eggs/day/female (Table 1). All *S*. *galapagense* accessions showed a reduction in OR and were significantly different from cv. Moneymaker. In fact there were three accessions on which no eggs were deposited. All *S*. *cheesmaniae* accessions were at least as susceptible as the cv. Moneymaker. At the species level, *S*. *galapagense* showed the lowest OR (OR = 1.3 ± 1.7, p < 0.001).

For several accessions it was impossible to determine PS, as there were no eggs deposited. For two accessions of *S*. *galapagense* (LA1452 and LA1401) we observed that, although whiteflies were able to lay a few eggs, none of those eggs hatched. The *S*. *cheesmaniae* accessions were not significantly different from the cv. Moneymaker in the PS values (Table 1).

### Trichome type and acyl sugar composition

A clear difference in the trichome composition was observed among the different tomato species. All accessions of *S*. *galapagense* had trichomes of both type I and IV. None of the accessions of *S*. *cheesmaniae* had trichomes type I or IV, while all of them had trichomes type V and VI in higher or lower densities (Additional file 1: Table S1). Trichome type III was absent on *S*. *galapagense* and present on 7 of the 22 accessions of *S*. *cheesmaniae* (Additional file 1: Table S1). A negative correlation between the measured resistance variables and the presence of glandular trichome types I, IV and VI was observed (Table 2).

**Figure 1:**
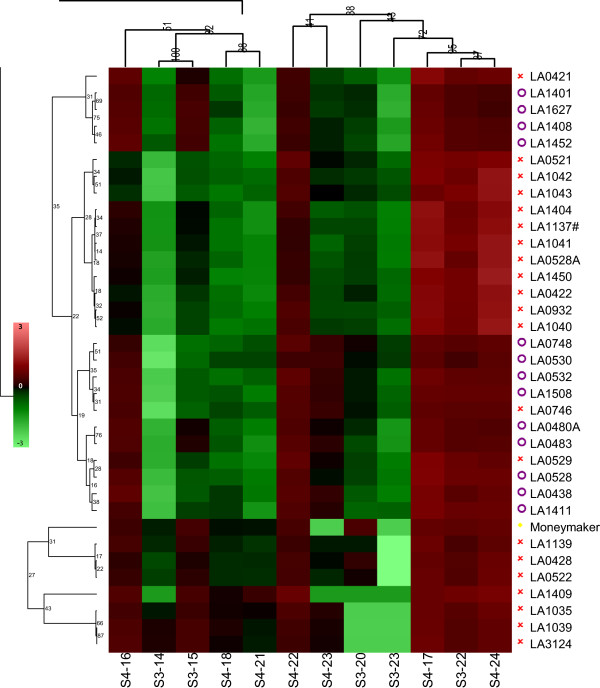
**Hierarchical clustering (Pearson correlation, UPGMA), of the different acyl sucroses (columns) and the different accessions (rows).***Solanum galapagense* S.C. Darwin & Peralta (violet circle symbol), *S*. *cheesmaniae* L. Riley (red cross sign symbol), *S*. *lycopersicum* L cv. Moneymaker (yellow rhombus symbol). Colour key is displayed in the figure.

**Figure 2:**
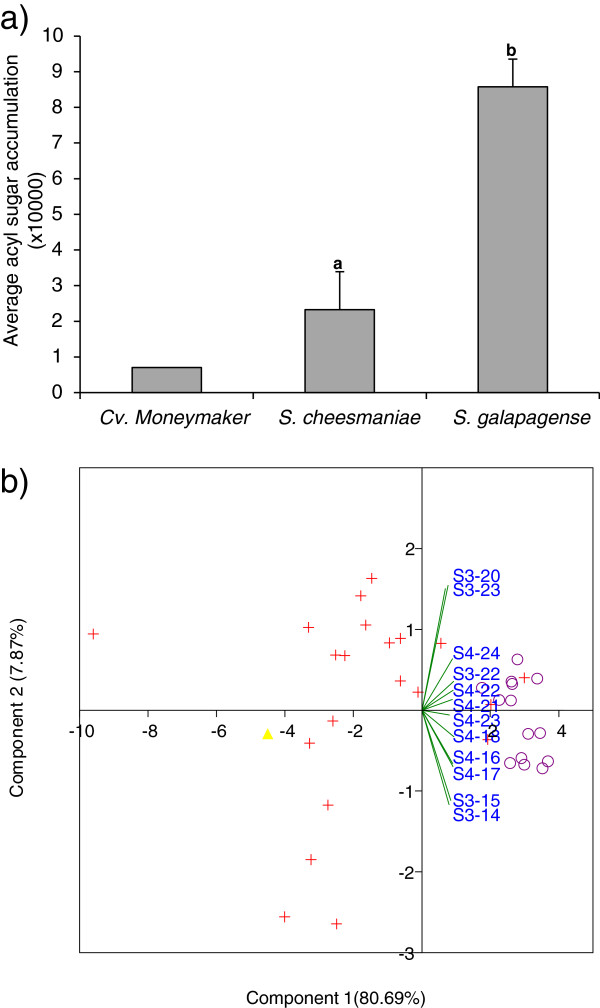
**Acyl sugar accumulation per species and accession. a)** Average acyl sugar accumulation per species; values for *S*. *galapagense* and *S*. *cheesmaniae* represent means and SD of 14 and 21 plants, respectively. Cultivar Moneymaker is included as reference. Different letters indicate statistical differences according to LSD test (p < 0.05); **b)** PCA-biplot score of the tomato accessions based on acyl sugar accumulation. *Solanum galapagense* (violet circle symbol, *S*. *cheesmaniae* (red plus sign symbol) and *S. lycopersicum* cv. Moneymaker (yellow rhombus symbol). Lines indicate the loadings of the different acyl sucroses.

The LC-MS analysis allowed us to determine the relative abundance of 12 acyl sugars. All of them were acyl sucroses: five with 3 lateral branches (S3-acyl sucroses) and seven with 4 lateral branches (S4-acyl sucroses). A heatmap (hierarchical clustering) visualizing the results is shown in Figure 1 and the relative amounts measured can be found in Additional file 1: Table S1. In Figure 1, we see two clusters: one including 7 accessions of *S*. *cheesmaniae* and the cv. Moneymaker and the other as an intermingled cluster with all the accessions of *S*. *galapagense* and the remaining accessions of *S*. *cheesmaniae*. Total acyl sugar abundance (expressed as the total of acyl sugar peak areas per accession) was higher in plants with a higher resistance level, measured as lower values for AS, OR and PS (R^2^ = 0.37, p < 0.001). In general, *S*. *galapagense* accessions accumulate higher levels of acyl sugars than *S*. *cheesmaniae* accessions. Nevertheless, there were four accessions of *S*. *cheesmaniae* (LA0421, LA0746, LA0521 and LA0529) with levels of acyl sucroses that were as high as those found in *S*. *galapagense* accessions (Figure 2, Additional file 1: Table S1). Regression analysis did not indicate a specific relation between acyl sugar accumulation, neither qualitatively nor quantitatively, and the type of trichomes present on a plant. However, we observed a slight positive correlation between total acyl sugars and the presence of glandular trichomes type I and IV (Table 2).

### Genetic relationships and correlations among accessions

**Figure 3:**
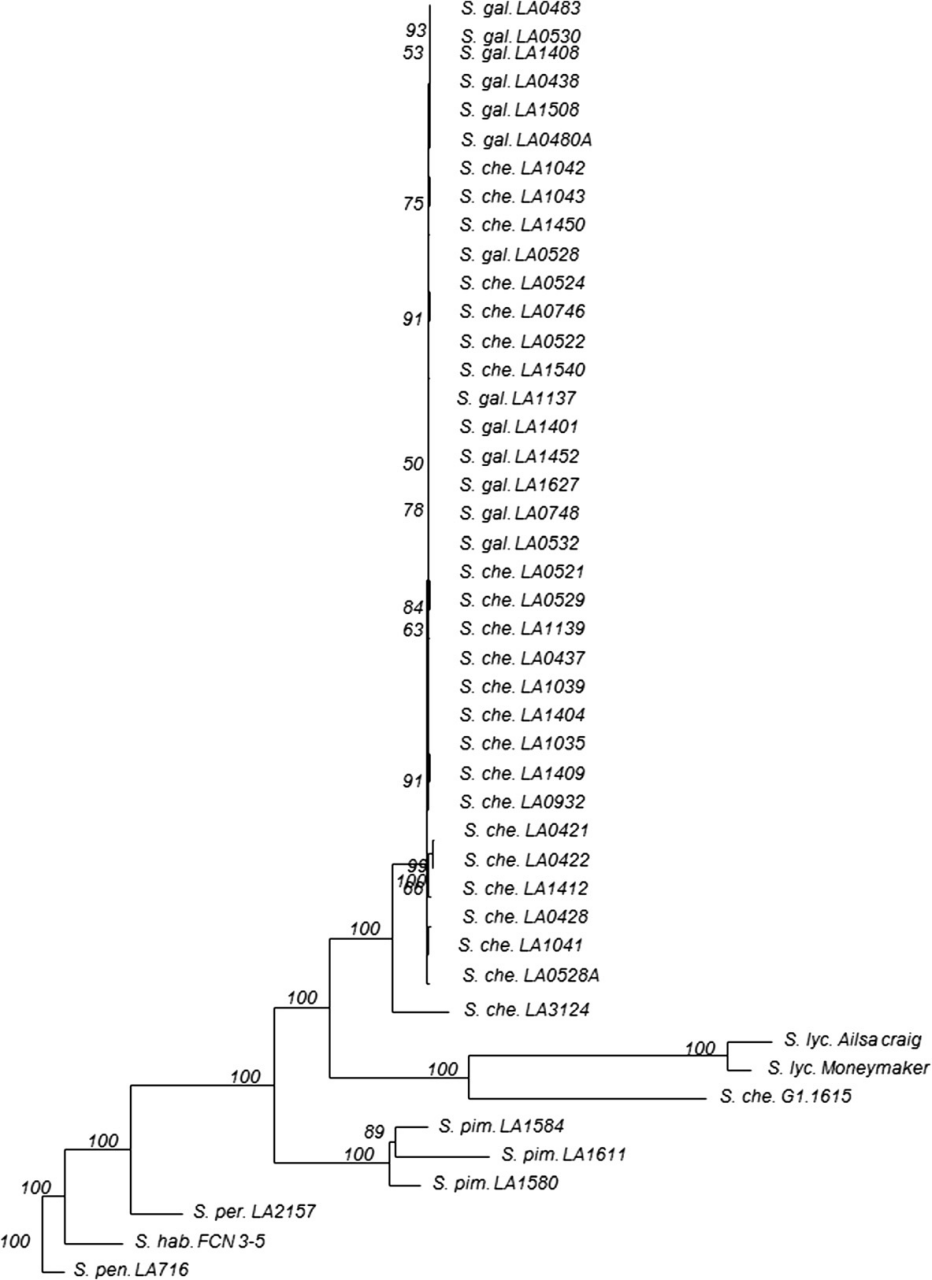
**Neighbour-joining tree of the accessions.** The NJ tree was based on Manhattan distances using 3324 polymorphic markers. Per sample the species name is followed by the accession code (*S*. *che*. for *Solanum cheesmaniae*; *S*. *gal*. for *S*. *galapagense*; *S*. *pim*. for *S*. *pimpinellifolium*; *S*. *lyc*. for *S*. *lycopersicum*; *S*. *per*. for *S*. *peruvianum*; *S*. *hab*. for *S*. *habrochaites* and *S*. *pen*. for *S*. *pennellii*). Bootstrap values (higher than 50) are shown on the branches.

We used a SNP array [23] to determine the genetic relationships between the different accessions. The Neighbour joining tree (Figure 3), based on 3316 markers that showed polymorphisms in or among *S*. *lycopersicum*, *S*. *pimpinellifolium*, *S*. *cheesmaniae* and *S*. *galapagense*, indicated a tight cluster with all the accessions of *S*. *galapagense* and *S*. *cheesmaniae* intermingled. Two accessions of *S*. *cheesmaniae* (LA3124 and G1.1615) were clearly separated from the rest and are most likely hybrids with either *S*. *lycopersicum* or *S*. *pimpinellifolium*. After exclusion of these two deviating *S*. *cheesmaniae* accessions only 53 polymorphic markers (Additional file 2: Figure S1) were detected within *S*. *galapagense* and *S*. *cheesmaniae*. From those 53 SNPs, 44 were polymorphic among *S*. *cheesmaniae* accessions and 9 among *S*. *galapagense* accessions. Most of the polymorphic markers were randomly distributed over the two species, 7 of them were found only in one accession and 2 were unique to *S*. *galapagense*. None of the polymorphic markers were fixed in either species.

### Correlation of species presence to geographic/climatic variables

**Figure 4:**
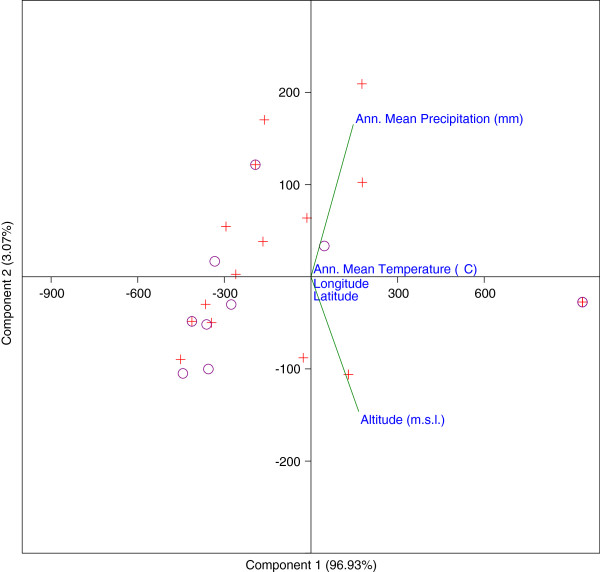
**PCA-biplot score of the accessions of *Solanum galapagense *(violet circle symbol) and *S*. *cheesmaniae *(red plus sign symbol) based on geographic/climatic variables.**

We analysed the available geographic/climatic data for the Galapagos Islands in relation to the location where the *S*. *galapagense* and *S*. *cheesmaniae* accessions were collected, but no correlation was detected (Figure 4).

## Discussion

### Whitefly resistance in *S*. *galapagense*, a combination of trichomes and metabolites

A difference in whitefly resistance was observed between *S*. *galapagense* (resistant) and *S*. *cheesmaniae* (susceptible). While some *Solanum* species are considered to be resistant, it is important to note that the level of resistance may vary between and within accessions of the same species. Next to resistance to whitefly [17], such inter- and intra-variation was also described, among others, in a screenings for white mold and late blight resistance in potato [17,24-26]. We have found resistance to whitefly in all *S*. *galapagense* accessions, which was accompanied by high densities of trichomes type IV and high acyl sugar accumulation. Based on available data and literature it is not known whether the original founder had type IV trichomes or not. Even though the accessions of *S*. *galapagense* differ in the relative amounts of the different acyl sugars present, they were all resistant. This was not the case for *S*. *cheesmaniae*. Although some accessions of *S*. *cheesmaniae* accumulated acyl sugars to levels comparable to those found in *S*. *galapagense* they were all susceptible, probably because they lack trichome type IV. Contrarily, we also observed that in some accessions of *S*. *pimpinellifolium*, although they had trichome type IV, the levels of acyl sugars and the resistance variables were not different from those found on cv. Moneymaker (data not shown). The role of glandular trichomes and acyl sugars in insect resistance has been discussed frequently [11,16,17,27,28]. In tomato, the synthesis and accumulation of acyl sugars takes place within the glandular head of the trichome [29]. Acyl sugars are non-specific resistance components providing resistance to a broad spectrum of insects of different feeding guilds (whiteflies, aphids, leaf miners, caterpillars, etc.) [30]. Based on our data it is likely that a minimum level of acyl sugars and the presence of glandular trichomes type IV are needed to achieve an effective level of resistance and a fully resistant phenotype. It also suggests that not a specific acyl sugar, but rather the total amount of acyl sugars is important for resistance. Having said so, it is possible that others metabolites, not detected by the LC-MS analysis, may be important as well.

### Galapagos’ endemic tomato species cannot be differentiated by genetic analysis

The clear morphological differences between *S*. *galapagense* and *S*. *cheesmaniae* with regard to their leaf morphology, trichome composition, internode length, among others, were the reason to consider them as two distinct species [1]. However, our SNP array analysis based on 5528 markers [23], of which 3316 were polymorphic among the Lycopersicon group of *Solanum* sect. *Lycopersicon*, showed only 53 polymorphisms within the two species, with only two alleles specific to *S*. *galapagense* and no fixed alleles. As to be expected also in the NJ tree the two species could not be separated. Similar observations have been made using AFLP markers [5]. The observed low genetic variation among accessions of the two species under study is consistent with the hypothesis that these endemic species are the result of a unique founder event on the Galapagos Islands, followed by morphological divergence. The very obvious morphological differences between *S*. *galapagense* and *S*. *cheesmaniae* might be due to a limited number of genetic changes that have not been picked up by our SNP array. For instance, a single nucleotide deletion in the promoter of the *PTS*/*TKD1* gene results in a marked change of leaf complexity as seen in *S*. *galapagense*[31]. *Solanum galapagense* and *S*. *cheesmaniae* are part of a monophyletic clade within *Solanum* sect. *Lycopersicon*, together with *S*. *pimpinellifolium* and *S*. *lycopersicum*[7]. Thus, based on the molecular marker analysis it might be more appropriate to consider them as morphotypes, rather than as different species. In line with this, hybrids between *S*. *galapagense* and *S*. *cheesmaniae* have been found [3]. In addition, we cannot ignore the clear morphological differences (trichomes, whitefly resistance, leaf morphology, etc.) found by us and by others [1]. All this suggest that the species status of these two tomatoes should be reconsidered carefully taking into consideration the different concepts used to define the species.

Biogeographic variables, phylogenetic studies and experimental approaches can be used to answer questions like adaptation to local environments, divergence and prediction of a phenotype based on the environment where a species was found [19,25,32-34]. We observed no correlation between the geographic location where the accession was collected and the occurrence of one of the two morphotypes. Some studies have addressed the population structure of wild tomatoes and external factors causing this. Caicedo and Schaal [35] observed a clear structure in *S*. *pimpinellifolium* populations collected from northern to southern Peru, which was consistent with a hypothesis based on genetic isolation by distance. It was also observed that genetically closely related accessions can be found far away from each other. In our analysis, we also saw a correlation between genetic distance and geographical distance (Additional file 2: Figure S1), however this has to be considered with caution as the low level of genetic variation may cause artefacts. Zuriaga *et al*. [36] extended the analysis of Caicedo and Schaal [35] by including accessions from Ecuador as well, and they suggested that the population structure could be better explained by ecological and climatic variables rather than by geographic distances. Similar results were obtained for populations of *Solanum habrochaites*[20,21] were geographical and climatic variables explain a substantial amount of the variation in terpenoids and acyl sugars. However, in our analysis, we did not see such correlation between climatically or geographical conditions at the collection sites of the accessions and the morphological species boundaries.

### Can selection pressure explain morphological differences?

Starting from the generally accepted assumption [18,37] that maintenance of a constitutive resistance mechanism like the one present in *S*. *galapagense* (i.e. trichomes, acyl sugars) is energetically expensive and thus only viable when there is a high selection pressure. One possible explanation to consider is that the resistance mechanisms found in *S*. *galapagense* serves other functions as well. In other words, resistance is a secondary function of a trait evolved in response to some other biotic or abiotic pressure [18,38]. For example, a leaf surface characteristic such as high trichome density may be a direct defence against insects or pathogens like bacteria. It may also be an indirect defence mechanism against viruses although no reports mention the occurrence of whitefly transmitted viruses in *S*. *galapagense* and/or *S*. *cheesmaniae*. Trichomes might be of even greater adaptive value against other environmental stresses such as water loss. Flanders et al. [38], working with potato accessions distributed from USA to Argentina, attributed the observed resistance patterns towards potato herbivores (potato aphid, Colorado potato beetle, potato flea beetle and potato leafhopper) to variation in geographical (altitude) and climatic conditions. Specifically, they found that accessions from hot and dry areas were more resistant to Colorado potato beetle and potato flea beetle. However, considering that *S*. *galapagense* and *S*. *cheesmaniae* are found in sympatry in space and time [3,5] and that within our set of accessions there was no significant difference between *S*. *galapagense* and *S*. *cheesmaniae* for any of the climatic/geographical variables, it is unlikely that maintenance of the resistance mechanisms could be explained by this hypothesis.

Another plausible explanation is that resistance traits in tomato (trichomes, secondary metabolites) are under selective pressure. It was observed that *S*. *galapagense* is more abundant and widespread in undisturbed areas than *S*. *cheesmaniae*[5]. Although it has being proven that trichomes and their exudates also have an effect against other pathogens [39], we have focused our analysis on whiteflies. Though, *B*. *tabaci* was only recently found on the Galapagos Islands (1998) [9], numerous other herbivores sharing the same feeding guilds (i.e. *Myzus persicae*, *Macrosiphum sp*.) are present [9,40]. Recently it was shown that even the selective pressure of a single insect herbivore species (either a generalist or a specialist) can be strong enough to shift the allele frequencies of a plant population within a few generations [41-43]. Züst *et al*. [43], working with *Arabidopsis* populations and aphids (a generalist phloem-feeder), reported that the trichome densities of *Arabidopsis* plants in presence of herbivores remains constant over generations and decrease in an herbivore free environment. The authors also proved that aphid populations had an effect on the frequency of the plant genotypes with different aliphatic glucosinolates. It was also proven that there was an indirect effect of herbivory selection pressure in the sense that some accessions were able to compete with others only in the absence of herbivores, showing the ecological benefits of having a resistance trait only in the presence of herbivores. Agrawal *et al*. [41] provided evidence that herbivory by a specialist insect pest (seed predator moth) can act as a direct selective force in favour of the resistance, but also as an indirect selective force to enhance competitive ability of the plant in the presence of herbivores. All this information provides evidence to support the hypothesis that the current resistance mechanisms present in *S*. *galapagense* could be maintained by selection pressure, rather than by geographical/climatic variables. This hypothesis can explain how it is possible for the plant to maintain a high level of morphological differentiation (resistance level, trichome composition) with a relatively low genetic variation and in the presence of gene flow.

## Conclusions

Our results show that whitefly resistance was found exclusively in *S*. *galapagense* accessions and that it was associated with the presence of type IV trichome and high levels of acyl sugars. Our marker and metabolomics data support the hypothesis that *S*. *galapagense* and *S*. *cheesmaniae* might be morphotypes rather than two species and that their co-existence is likely the result of a selective pressure.

## Methods

### Plant materials and growing conditions

**Figure 5:**
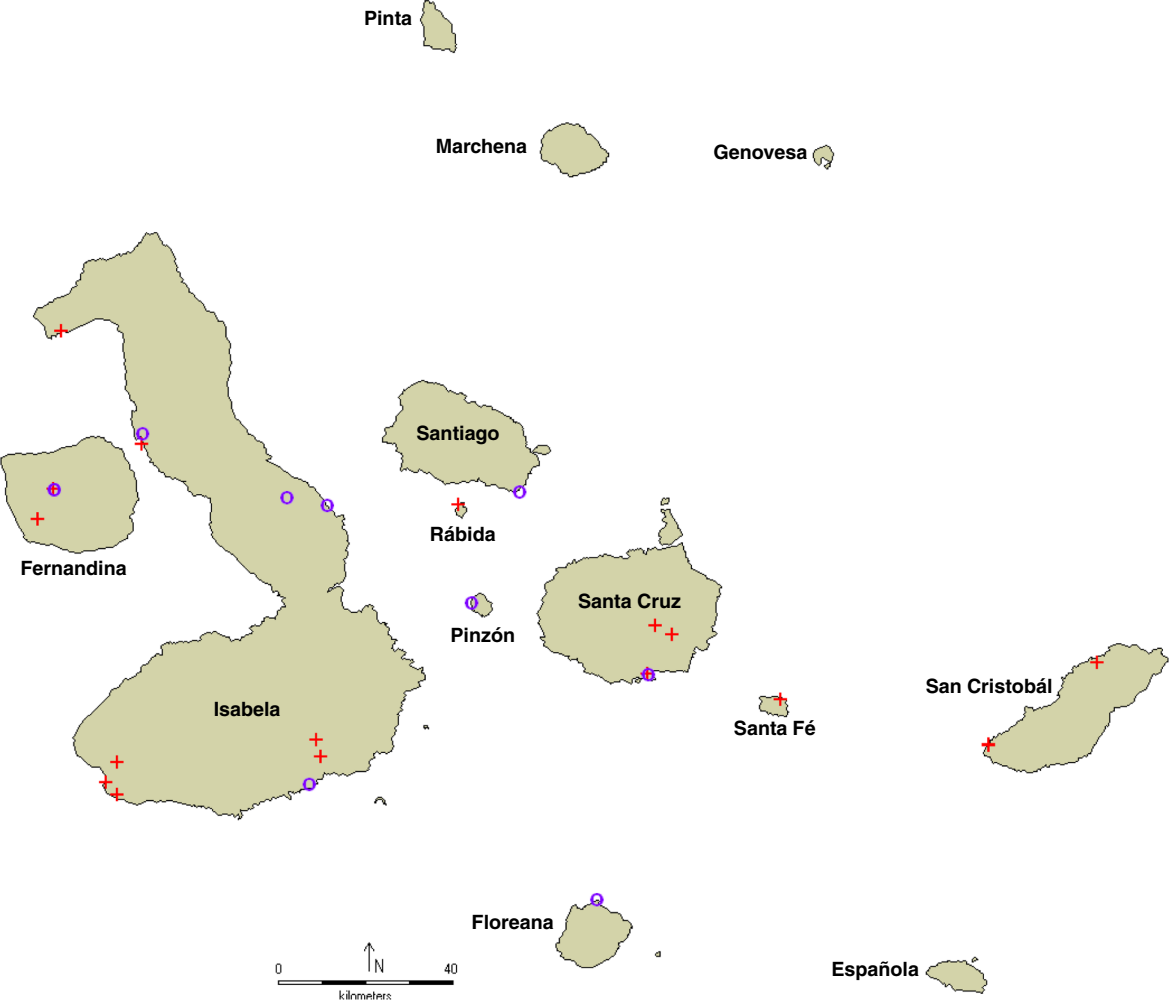
**Geographical distribution of the tomato accession on the Galapagos Islands.***Solanum galapagense* S.C. Darwin & Peralta (violet circle symbol); *S*. *cheesmaniae* L. Riley (red plus sign symbol**)**. Map made with DIVA-GIS version 7.5 [44].

In total, we evaluated 35 tomato accessions (Table 1, Additional file 1: Table S1), covering the geographical distribution as much as possible (Figure 5). These included 22 accessions of *Solanum cheesmaniae* L. Riley, 12 accessions of *S*. *galapagense* S.C. Darwin & Peralta, and 1 accession of *S*. *lycopersicum* L. as reference.

The accessions were grown in a greenhouse at Wageningen UR Plant Breeding, Wageningen, the Netherlands (20 ± 2°C, 70% RH, 16/8 h day/night) in 14 cm pots. The plants were fertilized twice a week and watered once a day.

When the plants were six weeks old, they were moved to an insect proof greenhouse. One week before infestation the greenhouse temperature was increased slowly (two degrees per day) from 20 till 27°C to allow plants to adapt to the higher temperature (27 ± 2°C, 70% RH, 16/8 h day/night).

### Insect rearing

A non-viruliferous whitefly rearing (*Bemisia tabaci* Group Mediterranean-Middle East-Asia Minor I) was maintained on the tomato cultivar Moneymaker for several generations at Wageningen UR Plant Breeding, Wageningen, The Netherlands. The initial inoculum was obtained from a permanent rearing at the Laboratory of Entomology, Wageningen UR, Wageningen, The Netherlands.

### No-choice test

Whiteflies were anesthetized using CO_2_ and four days old females were selected under a binocular microscope by the morphology of the abdomen. Five females were placed into a clip-on cage (2.5 cm in diameter and 1.0 cm in high). Three clip-on cages per plant and four plants per accession were used. The cages were placed on the first to third fully expanded leaf, counting from the top of the plant, thereby taking care not to break the plant trichomes when assembling the cages. Five days after infestation, the number of death and alive whiteflies, as well as the number of eggs was registered from that day on. The surviving whiteflies were removed from the leaves and the adult survival (AS) and oviposition rate (OR) were calculated according to Bas *et al*. [45]. Due to the fact that the hatching of the eggs was irregular in time, we could not assess the number of newly hatched insects per day in order to calculate the development period (DP). When almost no new whiteflies were seen, the number of empty pupae was recorded and the pre-adult survival (PS) calculated [45]. A complete randomize assay with four replicas per accession was used. Each replica consisted of the average value of three cages per plant (technical replica). The variables were analysed with a one-way ANOVA followed by a least significant difference (LSD) test [46]. An Arcsin (Sqrt) transformation was applied to the variables adult survival (AS) and pre-adult survival (PS), whereas an Sqrt (x + 1) transformation was applied to the variable oviposition rate (OR). All statistical procedures were performed using the statistical software package Infostat Professional (2010) Cordoba, Argentina.

### Trichome description

Trichomes present on the abaxial side of the leaf were classified according to type [47]. For an estimation of trichome density, the abaxial part of three leaflets was observed under the binocular microscope and a visual scale was used to describe it. The scale used was adapted from Simmons and Gurr [16] and consisted of four categories: 3, Abundant (>5 per mm^2^); 2, sparse (5–1 per mm^2^); 1, very sparse (<1 per mm2), and 0, absent.

### Genotyping and phylogenetic analysis

Genomic DNA was extracted from one randomly selected plant per accession as described by Fulton [48]. The DNA concentration was adjusted to 50 ng/μl. For marker analysis a custom made single nucleotide polymorphism (SNP) Infinium bead array was used [23]. On this array 5528 tomato SNPs were present. The marker analysis was performed using the protocol provided by Infinium and carried out by Service XS, Leiden, The Netherlands. Marker data obtained were filtered using the following criteria: 1) monomorphic markers were removed; 2) markers were deleted when the frequency of heterozygotes per marker was equal to or higher than 5%, and 3) markers were deleted when the frequency of no-calls (NC) was equal to or higher than 50%. We choose a threshold of 50% for the NC because they can be the result of either an amplification problem (true NC) or the presence of a different, informative allele (false NC). The latter situation occurs frequently with more distantly related species [23]. After filtering a total of 3316 markers were used in the analysis. A phylogenetic tree was reconstructed by Neighbour Joining, using the Manhattan distance (Additional file 1: Table S1). The reliability of the resulting dendrogram was assessed by bootstrap analysis with 1000 replications. The analysis was carried out using the software package PAST [49]. Isolation By Distance (IBD, version 3.23) [50] was used to analyse for presence of isolation by distance.

### LC-QTOF-MS analysis

Four plants per accession were used for the chemo-profiling. From each plant, one complete leaf (second fully expanded leaf from the top of the plant) was cut, placed into an aluminium envelope and immediately frozen in liquid nitrogen. Each sample was frozen with liquid nitrogen and ground to a fine powder and storage at -80°C until use. Extraction and analysis by Liquid Chromatography-Quadrupole Time of flight-Mass Spectrometry (LC-QTOF-MS) [51,52]. Four hundred mg of frozen leaf powder was put into a glass tube with 1.2 mL of methanol/formic acid solution (99.9% - 0.1%). The samples were mixed using vortex, sonicated for 15 min and centrifuged at 2500 rpm for 10 min. The supernatant was filtered using a 0.45 μm filter, injected (5 μl) using an Alliance 2795 HT instrument (Waters), separated on a Phenomenex Luna C18 (2) column (2.0 × 150 mm, 3 mm particle size) using a 5–95% gradient of acetonitrile in water (both acidified with 0.1% formic acid) in 45 min and then detected by a Water-Micromass QTOF Ultima MS with electrospray ionization in negative mode (*m*/*z* 80–1,500). Annotation of LCMS peaks corresponding to acyl sugars was done on their accurate masses as previously described in Firdaus et al. [22]. The Quanlynx tool of the Masslynx acquisition software was used to calculate the relative abundance (peak area) of the different type of acyl sugars for all samples, based on their specific mass and retention times. The three isoforms of S3-20 (I, II, III) and the four of S3-22 (III – VI), as described before in Firdaus et al. [22], were considered as single peaks for S3-20 and S3-22, respectively, due to partly overlapping chromatographic peaks of these isomeric compounds in many samples. The peak areas were LOG_2_ (× + 1) transformed and auto scaled to the mean. PCA-biplot was done with the software package PAST [49], and all other statistical analysis were performed with the software package GeneMaths XT (version 2.21; Applied Maths, Belgium).

### Analysis of geographical distribution and climate data

The collection site information (latitude and longitude) of the accessions was obtained from the Tomato Genetic Resource Centre (TGRC). Locations and climate data were obtained from the WorldClim at 2.5 arc-min resolution [53] and were analysed as proposed by Gonzales-Vigil et al. [20], and using DIVA-GIS [44]. A regression analysis was carried out between the resistance variables (AS, OR, PS), the metabolites and the climatic/geographical variables (Altitude, Latitude, Longitude, Ann. Mean precipitation, Ann. Mean temperature).

## Competing interests

The authors declare that they have no competing interests.

## Authors’ contributions

The project was conceived by AFL, AWvH and BV. AFL performed the experiments and data analysis. RCHdV has been responsible for the LC-MS analyses and the data processing. AFL, AWvH, BV, RCHdV and RGFV have been involved in the writing of the manuscript. All authors read and approved the final manuscript.

## Supplementary Material

Additional file 1: Table S1 This file includes GeneBank origin, accession numbers, collection sites and all phenotypic information, SNP array data and LC-MS data for the material used in this work. The Phylogenetic tree was uploaded to TreeBASe.org, and can be found by following this link: http://purl.org/phylo/treebase/phylows/study/TB2:S14512. Click here for file

Additional file 2: Figure S1 Isolation by distance analysis. This figure describes the relation between the Genetic distances and the Geographic distances. Click here for file
