# Supplementary figures and images for: Differences in insect resistance between tomato species endemic to the Galapagos Islands
Source: BMC Evol Biol. 2013 Aug 24;13:175. doi: 10.1186/1471-2148-13-175 (PMC3765935; doi:10.1186/1471-2148-13-175)

## Slide 1
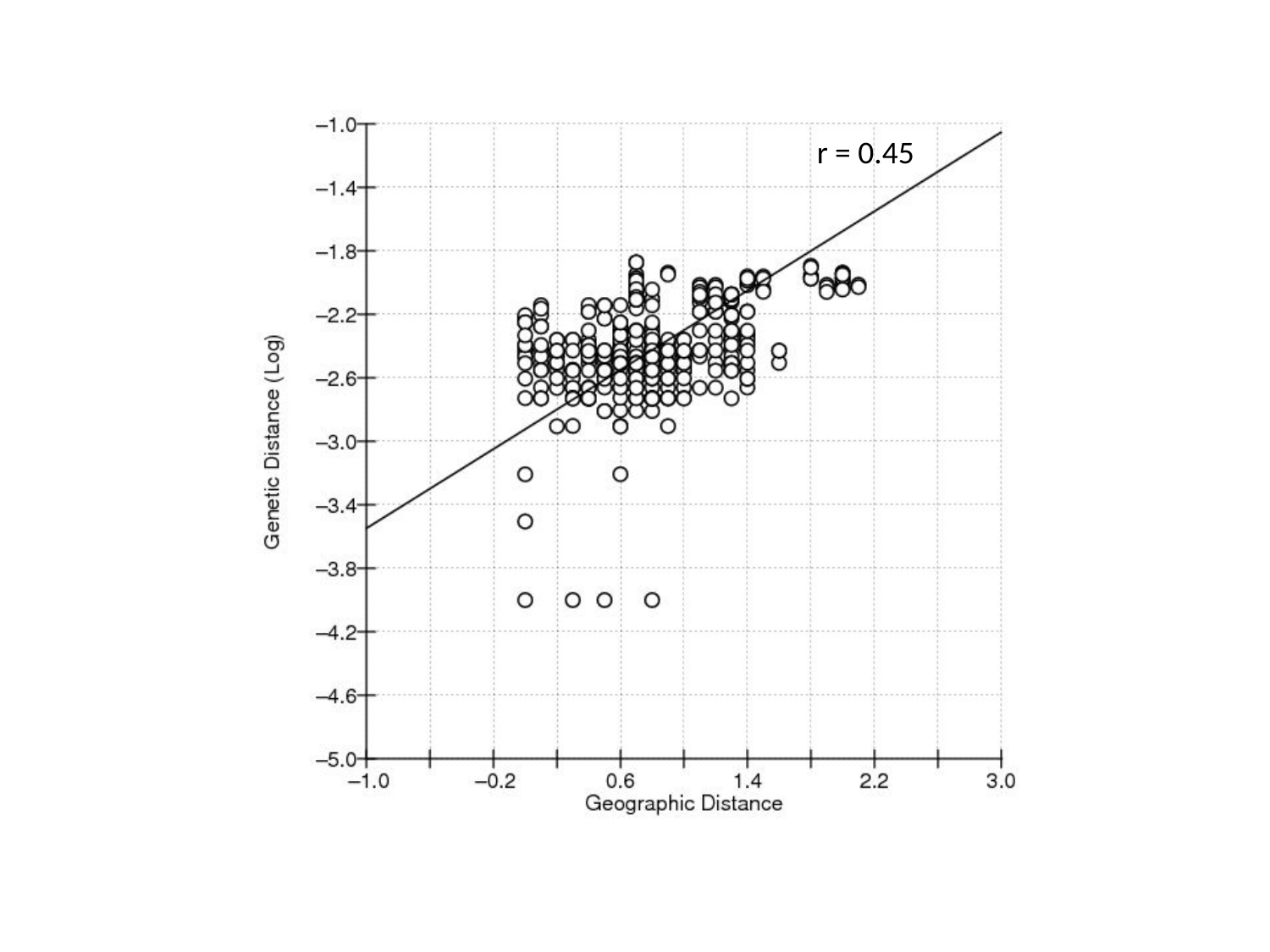

r = 0.45

Supplement: Additional file 2: Figure S1 — Isolation by distance analysis. This figure describes the relation between the Genetic distances and the Geographic distances. [file 1471-2148-13-175-S2.ppt]
